# Dynein Regulates Epithelial Polarity and the Apical Localization of *stardust A* mRNA

**DOI:** 10.1371/journal.pgen.0040008

**Published:** 2008-01-18

**Authors:** Sally Horne-Badovinac, David Bilder

**Affiliations:** Department of Molecular and Cell Biology, University of California Berkeley, Berkeley, California, United States of America; Huntsman Cancer Institute, United States of America

## Abstract

Intense investigation has identified an elaborate protein network controlling epithelial polarity. Although precise subcellular targeting of apical and basolateral determinants is required for epithelial architecture, little is known about how the individual determinant proteins become localized within the cell. Through a genetic screen for epithelial defects in the *Drosophila* follicle cells, we have found that the cytoplasmic Dynein motor is an essential regulator of apico–basal polarity. Our data suggest that Dynein acts through the cytoplasmic scaffolding protein Stardust (Sdt) to localize the transmembrane protein Crumbs, in part through the apical targeting of specific *sdt* mRNA isoforms. We have mapped the *sdt* mRNA localization signal to an alternatively spliced coding exon. Intriguingly, the presence or absence of this exon corresponds to a developmental switch in *sdt* mRNA localization in which apical transcripts are only found during early stages of epithelial development, while unlocalized transcripts predominate in mature epithelia. This work represents the first demonstration that Dynein is required for epithelial polarity and suggests that mRNA localization may have a functional role in the regulation of apico–basal organization. Moreover, we introduce a unique mechanism in which alternative splicing of a coding exon is used to control mRNA localization during development.

## Author Summary

Cells within epithelial sheets are highly polarized with distinct apical and basolateral membrane domains. This cellular organization is critical to both epithelial form and function, and a failure to maintain epithelial polarity is often linked to tumor progression. The protein network that establishes and maintains the two membrane domains relies on the precise subcellular localization of its molecular components, but little is known about how these proteins are targeted to their sites of action. We have shown that the localization of the apical determinant protein Stardust depends on the microtubule motor Dynein. While investigating the relationship between Dynein and Stardust, we also made two unexpected observations about *stardust* mRNA regulation. First, the mechanism by which Dynein localizes Stardust may depend, in part, on the apical targeting of the *stardust* mRNA. Second, some *stardust* mRNA is apically localized during early stages of epithelial development, but the selective removal of the apical localization signal leads to the sole production of uniformly localized transcripts in mature epithelial cells. Together, these results introduce roles for Dynein in apico–basal polarity regulation and raise important questions about the role of mRNA localization in the targeting of polarity determinant proteins and epithelial maturation.

## Introduction

A polarized architecture increases the level of sophistication at which cells can interact with their neighbors and the environment. Thus, it is not surprising that highly polarized epithelia have become the predominant tissue type among metazoans. Epithelial cells have distinct apical and basolateral membrane domains, each of which contains a unique complement of lipids and proteins to regionalize cellular functions. Intracellular junctions separate the apical from basolateral domains and mediate cell–cell adhesion and selective permeability within the monolayer. Although the mechanisms that specify apico–basal polarity have been a topic of great interest in recent years, there are still many open questions about how this cell architecture is established and maintained.

Studies in model organisms and mammalian tissue culture have identified an elaborate protein network regulating epithelial polarity across animal species [1–3]. At the core of this network are three protein complexes, which sequentially localize to and specify one of the two membrane domains and position the adherens junctions at their interface. The Baz complex (Bazooka/aPKC/Par6) acts first in the hierarchy to specify the apical domain [4–7]. The Scrib complex (Scribble/Discs Large) then functions as a basolateral determinant by repressing the apicalizing activity of the Baz complex. Finally the Crb complex (Crumbs/Stardust/DPATJ) is targeted to the apical membrane to antagonize the Scrib complex [6,7].

Crb is the only transmembrane protein among the known core polarity determinants and is a potent regulator of apical identity. Over-expression of this protein is sufficient to cause an expansion of the apical membrane at the expense of the baso-lateral domain in *Drosophila* [8]. Crb is stabilized in the plasma membrane by the cytoplasmic scaffolding protein, Stardust/MPP5 (Sdt), which binds Crb's cytoplasmic tail through its PDZ (PSD-95/Discs large/ZO-1) domain [9–12]. A third member of the complex, DPATJ, interacts with Sdt via shared L27 domains [12,13]. The Crb complex is highly conserved both at the structural and functional level between flies and vertebrates [12,14,15], and mutations in human *CRB1* have been linked to two severe forms of retinal dystrophy [16].

The Crb complex must be precisely localized to the apical domain to exert its effect on epithelial polarity, yet little is known about how the individual proteins within this complex are targeted to this position. Extensive studies in cultured mammalian cells have implicated exocytic vesicle trafficking in polarity regulation [17,18]. Under this paradigm, vesicles carrying newly synthesized transmembrane proteins are specifically targeted to either the apical or basolateral domain. While direct vesicle trafficking is an appealing potential mechanism for the localization of Crb, it is likely to be of limited use in the apical targeting of Sdt and DPATJ, the cytoplasmic members of this complex. Moreover, the currently recognized members of the Baz and Scrib complexes are entirely comprised of cytoplasmic proteins, which must be specifically localized to either the apical or lateral domains to perform their polarity functions. Uncovering the mechanisms that govern the subcellular targeting of cytoplasmic polarity determinants is critical, therefore, to our understanding of apico–basal polarity regulation.

One mechanism that many cell types employ to position proteins within the cytoplasm involves the subcellular localization of their corresponding mRNAs [19–24]. There are several strategies by which mRNAs can become enriched within a given cytoplasmic domain, but the most common strategy involves the assembly of transcripts into large RNA-protein particles that are transported along cytoskeletal elements by molecular motors. Subcellular transport, followed by localized translation, ensures that new proteins will only be produced in the cytoplasmic region where their function is required [25]. This mechanism, which is conserved across such diverse taxa as fungi, plants and animals, has been particularly well studied in the *Drosophila* oocyte where mRNAs encoding patterning regulators such as *oskar*, *gurken* and *bicoid* are precisely targeted to the three different regions of the oocyte where their protein products are required [23]. mRNA localization has also been observed in the *Drosophila* blastoderm, where transcripts such as *wingless*, and those encoding pair-rule genes, are targeted to the apical cytoplasm [26–29].

Here we show that loss of the minus end-directed microtubule motor Dynein in the *Drosophila* follicle cell epithelium leads to defects in apico–basal polarity. Our data indicate that Dynein is required for the localization of Sdt and suggest a mechanism whereby the Dynein-dependent apical targeting of specific *sdt* mRNA isoforms enriches Sdt protein in the apical cytoplasm. Surprisingly, the apical localization signal for the *sdt* mRNA maps to an alternatively spliced coding exon. Selective removal of this exon coincides with a developmental switch between localized and unlocalized isoforms of the transcript, in which apically localized *sdt* transcripts play an early role in the assembly of the Crb complex, while unlocalized transcripts are sufficient to maintain polarity in mature epithelia. These results suggest that the strategy for the apical targeting of the Crb complex changes during epithelial maturation and introduce a new class of gene products, mRNAs, to the existing paradigm for apico–basal polarity regulation

## Results

### Cytoplasmic Dynein Function Is Required for Epithelial Cell Shape and Integrity

**Figure 1:**
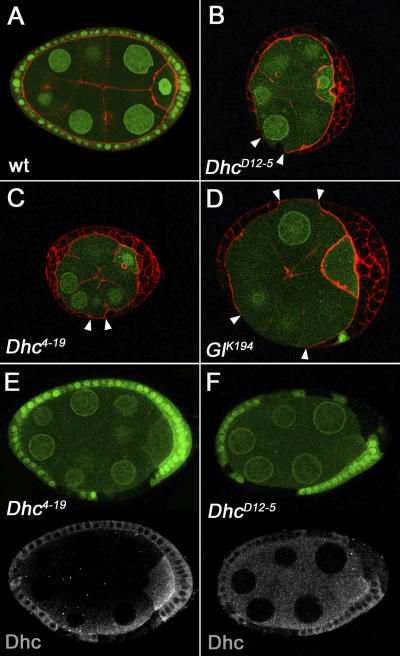
Cytoplasmic Dynein Function Is Required for Epithelial Cell Shape and Integrity (A–F) Wild-type cells are marked with green fluorescent protein (GFP). (A–D) Phalloidin staining (red) reveals FC morphology. (A) Wild-type ECs are surrounded by a uniform monolayer of cuboidal FCs. (B and C) Loss of Dhc function in the FCs, through either the newly isolated *D12–5* allele (B) or the previously characterized *4–19* allele (C), leads to disruptions of cell shape and gaps in the epithelium (arrowheads). (D) *K194*, a newly isolated allele of the Dynactin component *Glued*, phenocopies the loss of Dhc in the FCs. (E) Immunohistochemistry on mosaic egg chambers containing *Dhc^4^* ^−*19*^ mutant clones shows that Dhc protein levels (grayscale) are strongly reduced. (F) Dhc levels are similarly reduced in *Dhc^D12^* ^−*5*^ mutant clones.

To identify new genes required for epithelial architecture and morphogenesis, we performed a genetic screen in the follicle cell (FC) epithelium of the *Drosophila* egg chamber (EC). The FCs initially form a uniform, cuboidal epithelium surrounding the germ cells (Figure 1A), but undergo dramatic rearrangements during mid-oogenesis to create a columnar epithelium in the posterior of the EC and a squamous epithelium in the anterior [30]. We used EMS mutagenesis and the Flp/FRT system, under the control of the *e22c* Gal4 driver*,* to screen for epithelial defects in genetically mosaic FC epithelia. Mutant clones were identified by the lack of green fluorescent protein (GFP) and stained with rhodamine phalloidin to reveal cell shape. We screened approximately 5000 chromosomes and recovered 61 mutations that disrupt various aspects of epithelial morphology (S. Horne-Badovinac and D. Bilder, unpublished data).

Among the many phenotypes identified in the screen, we found two complementation groups, one with 3 alleles (*D12–5*, *N22–1*, *Q43–4*) and one with a single allele (*K194*), that showed identical phenotypes. Each of the four mutations causes rounding and multilayering of the FCs predominantly in the posterior of the EC as well as epithelial gaps, which likely arise from reduced cell-cell adhesion (Figure 1B and 1D, and data not shown). This combination of phenotypes is similar to those seen when either *crb* or *sdt* are mutated in the FCs ([31], unpublished data) suggesting that these new mutations might also disrupt apico–basal polarity by affecting the specification of the apical membrane domain.

We employed deficiency mapping, followed by candidate gene analysis, to identify the genes disrupted by these new mutations. All three alleles of the first complementation group failed to complement *Df(3L)Exel6102*. Complementation tests with known lethal mutations in this chromosomal region revealed that this group corresponds to *Drosophila's* lone cytoplasmic Dynein heavy chain gene, *Dhc64C.* Moreover, the second group failed to complement *Glued^1^*, a mutation that disrupts the p150 subunit of Dynactin, which functions as an accessory complex for Dynein transport [32]. Together these data show that both complementation groups disrupt genes required for cytoplasmic Dynein transport and indicate that this motor is required for cell architecture and epithelial continuity in the FC epithelium.

It is curious that our screen identified three alleles of *Dhc64C*, as it had been previously reported that this gene is required for cell viability in somatic clones [33]. To provide further evidence that this complementation group does indeed disrupt *Dhc64C*, we made FC clones that were homozygous for *Dhc^4^* ^−*19*^, a previously characterized strong *Dhc* allele [33]. *Dhc^4^* ^−*19*^ mutant FC clones are not only viable, but contain rounded, multi-layered cells and epithelial gaps highly similar to mutant clones for our newly isolated alleles (Figure 1C). Moreover, in both *Dhc^4^* ^−*19*^ and *D12–5* mutant clones, Dhc protein levels are significantly reduced when compared to wild-type cells (Figure 1E and 1F). Finally, introduction of a *Dhc* transgene was sufficient to rescue lethality of *D12–5* hemizygotes (data not shown). From these data we conclude that *D12–5*, *N22–1* and *Q43–4* are bona fide *Dhc* alleles and that our ability to make strong *Dhc* loss-of-function FC clones provides a unique opportunity to study cytoplasmic Dynein function in epithelia.

### Loss of Dhc Disrupts the Apical Localization of Sdt and Crb

**Figure 2:**
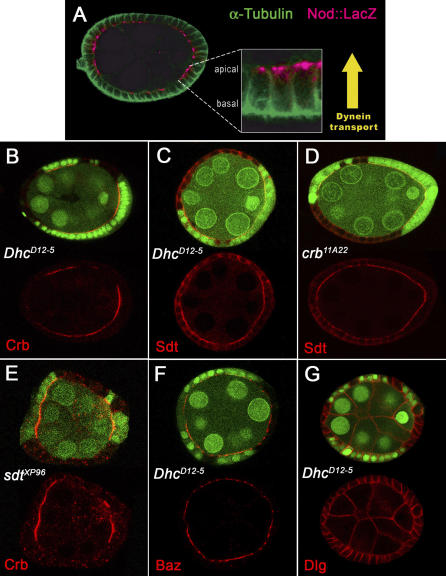
Loss of Dhc Disrupts the Apical Localization of Sdt and Crb (A) An anti-α-Tubulin antibody (green) reveals MTs in the FCs, while a *nod::LacZ* transgene (magenta) shows that the MT minus ends point toward the apical surface. Dynein is known to transport cargo toward MT minus ends. (B–G) Wild-type cells are marked with GFP (green) in mosaic egg chambers. (B) Crb is virtually absent in *Dhc* clones. (C) Sdt is largely missing from the apical surface in *Dhc* clones and is instead found throughout the cytoplasm. Mis-localization of Sdt in *Dhc* clones cannot be due solely to the loss of Crb, as *crb* clones consistently retain a punctate Sdt signal near the apical side of the cell (D). Mislocalization of Sdt in *Dhc* clones could account for the loss of Crb, however, as Crb is strongly disrupted in *sdt* clones (E). (F) Baz is only slightly reduced at the apical surface in *Dhc* cells. (G) Dlg localization is largely normal in *Dhc* clones*.*

How does loss of Dhc lead to cell architecture defects in the FCs? Dhc is the force-generating subunit of the cytoplasmic Dynein motor, which transports diverse cargoes, such as vesicles, mRNAs and organelles, toward microtubule (MT) minus ends in eukaryotic cells. As is typical for polarized epithelia, FCs contain a dense population of MTs along their lateral cortex with their minus ends pointed toward the apical surface (Figure 2A) [34,35]. This cytoskeletal geometry suggests that Dynein could affect cell architecture by targeting apical determinant proteins to their site of action at the apical cell surface. Crb stands out as a potential Dynein cargo because it is the only transmembrane protein among the known apical determinants. We initially hypothesized, therefore, that Dynein might affect apico–basal polarity by transporting vesicles containing newly synthesized Crb to the apical surface.

To test whether Dynein is required for the apical targeting of Crb, we performed immunohistochemistry on *Dhc* FC clones and focused our analysis on lateral clones, in which mutant cells retain cuboidal morphology. The results show that, even in *Dhc* cells with relatively normal shape, Crb is missing from the apical surface (Figure 2B). Although *Dhc* cells occasionally display a weak, cytoplasmic staining for Crb (data not shown), overall protein levels are severely reduced when compared to wild-type cells. These experiments show that Dynein is indeed required for the apical targeting of Crb and suggest that Dynein transport is also necessary for the maintenance of wild-type Crb levels in the FCs.

The fact that Crb is missing from the apical surface in *Dhc* clones is consistent with our initial hypothesis that Dynein could be transporting Crb-containing exocytic vesicles, but we were struck by the strong general reduction in Crb protein levels. It has been previously shown that Crb levels are reduced in embryonic epithelia when its cytoplasmic binding partner *stardust (sdt)* is mutated [36], so we next investigated whether Dynein is required for Sdt localization. Sdt is strongly depleted from at the apical surface in *Dhc* cells and is instead found throughout the cytoplasm (Figure 2C). These data indicate that Dynein is also required for the apical targeting of Sdt and suggest that the mechanism by which Dynein localizes Crb could be indirect.

Although it is well established that Crb and Sdt depend upon one another for their apical localization, the degree of this dependency can vary with the identity and developmental stage of the tissue examined [9,10,36]. To determine whether the disruption of just one of these proteins in *Dhc* FC clones could account for the mis-localization of the other, we investigated the relationship between Crb and Sdt in pre-vitellogenic egg chambers. Crb is only partially required for the apical localization of Sdt in the FCs, as there is still a significant amount of Sdt at the apical surface and cell junctions of *crb* mutant cells prior to stage 8 (Figures 2D and S1A). The Sdt localization defect in *crb* clones is, therefore, much weaker than that of *Dhc* clones. However, Crb protein is missing from the apical surface of *sdt* clones from the very earliest oogenic stages and overall Crb protein levels are strongly reduced (Figures 2E and S1B), a phenotype indistinguishable from that of *Dhc* clones. Together these data indicate that that the mislocalization of Sdt in *Dhc* clones can potentially account for the loss of Crb.

Is Dynein's role in Crb and Sdt localization specific or does loss of Dynein lead to a general disruption of protein polarization? This question is particularly important because a recent study showed that Dynein helps localize Baz to the apical surface in the cellularizing embryonic blastoderm [5]. In contrast to the strong reductions in Crb and Sdt, *Dhc* FC clones show only modest reductions of Baz, aPKC and Par6 at the apical surface (Figures 2F and S2). Because Crb is missing in *Dhc* mutant cells we investigated whether Baz complex components were similarly reduced in *crb^11A22^* cells and found a nearly identical phenotype (Figure S2). These results suggest that the apical reduction of Baz in *Dhc* clones may be secondary to the loss of Crb. It is likely that Dynein does play some role in the apical targeting of Baz in the FCs, however, as we did observe rare *Dhc* clones in which Baz and aPKC were relocalized from the apical to the lateral domain, a phenotype never seen in *crb* clones (Figure S2). Finally, the apical localization of β_H_-Spectrin is indistinguishable from wild-type (unpublished data), as is the localization of Discs-large (Dlg) to the lateral domain (Figure 2G). In sum, these protein localization experiments indicate that Dynein is required for the proper targeting of a subset of apical proteins in the FCs and that it is particularly important for the localization of Sdt.

### *sdt* Genetically Interacts with *Dhc64c*

**Figure 3:**
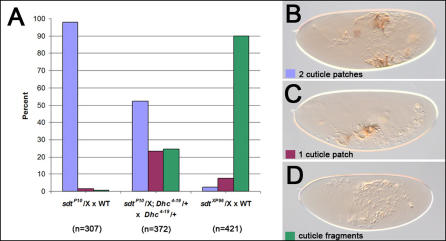
*sdt* Genetically Interacts with *Dhc64C* (A) Graph quantifying the strong genetic interaction between *sdt* and *Dhc64C,* demonstrated by the cuticle phenotypes expressed by *sdt* embryos from three different genetic crosses. *sdt^P10^* is a hypomorphic allele, which typically displays two patches of cuticle in addition to small cuticle fragments (B). In the strong *sdt^XP96^* allele, the epidermis only produces small cuticle fragments (D). When Dhc levels are reduced in *sdt^P10^* mutants, the cuticle in many embryos is reduced to a single patch (C) or strongly reduced to fragments, like *sdt^XP96^* (D).

The molecular epistasis experiments above suggested a clear relationship between Dynein and Sdt in FC apico–basal polarity regulation. Since Sdt is a central regulator of embryonic epithelial polarity, we next wanted to determine whether this association also exists in embryos. However, unlike the FCs, it is not possible to study Dynein under strong loss of function conditions in *Drosophila* embryos due to its large maternal contribution and essential function in oogenesis [33,37]. We therefore looked for a genetic interaction between *sdt* and *Dhc64C* in embryos by asking whether reducing Dynein levels in a hypomorphic *sdt* mutant background could further impair Sdt activity. *sdt^XP96^* is a strong allele that severely reduces cuticle production, resulting in only small cuticle fragments in almost all embryos (Figure 3D). By contrast, embryos hemizygous for the hypomorphic allele, *sdt^P10^*, typically have two large cuticle patches (Figure 3B). We impaired Dynein function in this background by crossing the strong *Dhc^4^* ^−*19*^ allele to *sdt^P10^* (see Methods for experimental details). Embryos collected from the cross in which the *sdt^P10^* and *Dhc^4^* ^−*19*^ alleles were both segregating showed reduced cuticle production compared to *sdt^P10^* mutants alone. In 48% of these embryos, the cuticle was reduced to either a single patch (Figure 3C) or small fragments, similar to *sdt^XP96^* mutants (Figure 3A). The clear genetic interaction between *sdt* and *Dhc64C* indicates that the apical localization of Sdt is likely to depend on Dynein in both FC and embryonic epithelia.

### The Apical Localization of *sdt* mRNA Is Dynein-Dependent and Developmentally Regulated

How does Dynein localize Sdt to the apical surface? Although Dynein could in theory be trafficking Sdt protein directly, this motor can also localize proteins indirectly by transporting the transcripts that encode them [21,23,24]. A single, localized mRNA molecule can generate many proteins in the desired location, making mRNA transport a highly efficient mode of protein localization. Because *sdt* transcripts have been reported to be apically localized in embryonic epithelia [9], we investigated whether Dynein might localize the Sdt protein through the apical targeting and localized translation of its mRNA.

**Figure 4:**
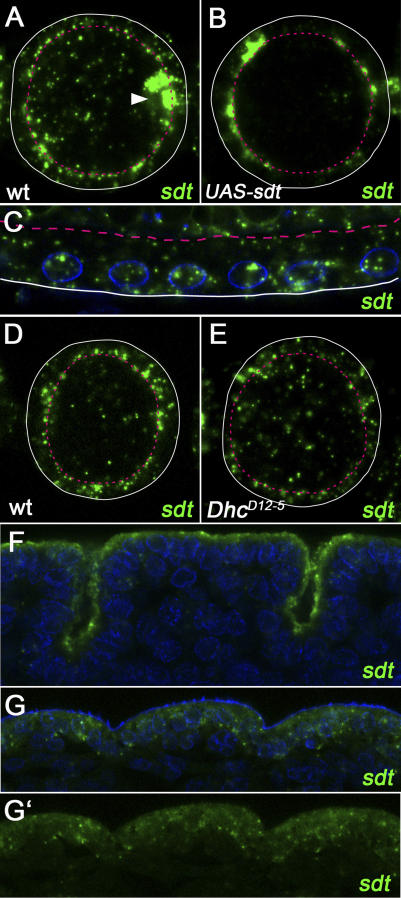
The Apical Localization of *sdt* mRNA Is Dynein-dependent and Developmentally Regulated Fluorescent in situ hybridization (green) reveals the localization of the *sdt* mRNA in the FCs (A–E) and embryonic surface ectoderm (F and G). (A–E) Pink lines denote the estimated position of the apical surface, and white lines mark the basal surface. (C), (F and G) Nuclear envelopes are stained with wheat germ agglutinin (WGA, blue). (A) At stages 2–4, *sdt* mRNA forms a punctate ring at the apical FC surface. An arrowhead points to *sdt* mRNA localization in the germline, which is better shown in Figure S3. (B) Over-expression of the *sdt cMAGUK* transgene in the FCs reveals strong apical transcript localization. (C) At stage 10, *sdt* mRNA is uniformly distributed in the FC cytoplasm. The apical ring of sdt puncta normally observed in wild-type egg chambers (D) disappears when the entire epithelium is mutant for *Dhc^D12^* ^−*5*^ (E). (F) At embryonic stage 11, *sdt* transcripts are strongly enriched in the apical cytoplasm, but appear to be uniformly distributed by stage 15 (G and G')

To determine whether *sdt* mRNA is apically localized in the FCs, we performed fluorescent in situ hybridization on ovaries. During oogenic stages 2–4, *sdt* transcripts appear as small puncta that are greatly enriched in the apical FC cytoplasm (Figure 4A). This apical localization is particularly evident when a *sdt* transgene (*sdt cMAGUK*,[9]) is over-expressed (Figure 4B). Surprisingly, however, *sdt* transcripts are uniformly distributed in the FC cytoplasm during later oogenic stages (Figure 4C). These data indicate that *sdt* mRNA is apically localized in the FCs, but that transcript localization can be developmentally regulated. Interestingly, we also found a similar developmental switch in embryos: *sdt* transcripts are predominantly apical during the first half of embyogenesis (Figure 4F), but become randomly localized at later stages (Figure 4G). That *sdt* mRNA localization is similarly regulated in both FC and embryonic epithelia suggests that a developmental switch in *sdt* mRNA localization may correlate with epithelial maturation in *Drosophila*.

We then took advantage of our ability to make strong loss of functions clones in the FCs to investigate whether Dynein is required for the apical enrichment of *sdt* transcripts during early oogenic stages. When compared to the ring of apical *sdt* puncta observed in wild-type FCs (Figure 4D), *sdt* puncta are randomly distributed in FC epithelia composed entirely of *Dhc* mutant cells (Figure 4E). To quantify this effect, we counted *sdt* mRNA puncta in each of three compartments: the nucleus, the apical cytoplasm or the basal cytoplasm. Among cytoplasmic puncta in wild-type cells, 74% were apical (*n* = 8 ECs). In *Dhc* FCs, however, only 44% of cytoplasmic puncta were apical (*n* = 7 ECs). The t value for this data set is 13.49 indicating statistical significance (Figure S4; Table S1). These data show that Dynein is required to enrich *sdt* transcripts in the apical FC cytoplasm during the same period when epithelial polarity defects are first seen in *Dhc* FC clones.

### The *sdt* Apical Localization Signal Is Located in an Alternatively Spliced Coding Exon

**Figure 5:**
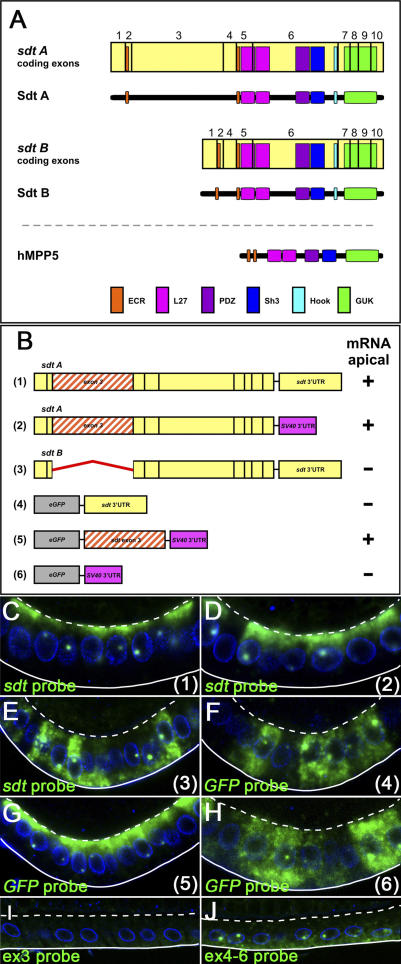
Exon 3 Contains the *sdt* Apical Localization Signal (A) Diagram showing the relationship between the exon structure of two *sdt* mRNA isoforms and the protein domains they encode. Exon 3, which is deleted from the *sdt B* isoform, encodes no conserved functional domains. The stretch of amino acids encoded by exon 3 is not conserved in other Sdt homologs such as human MPP5. (B) Diagram summarizing the experimental results from the six UAS-transgenes used to map the *sdt* apical localization signal to exon 3. All transgenes were expressed under the control of the GR1 FC driver. (C–H) Fluorescent in situ hybridization (green), using probes against either *sdt* (C–E) or *GFP* (F–H) reveals the localization of transgenic mRNAs in the FCs at stages 8–9. (C) Transcripts from a transgene representing the *sdt A* isoform localize apically. (D) *sdt A* transcripts continue to localize apically without the *sdt* 3′UTR. (E) Transcripts from a transgene representing the *sdt B* isoform, which lacks exon 3, are distributed uniformly in the cytoplasm. (F) Adding the *sdt* 3′UTR to an *eGFP* transgene is insufficient to target these transcripts apically. (G) Adding *sdt* exon 3 to an *e*GFP transgene is sufficient to send the mRNA apical. (H) Neither the *SV40* 3′UTR nor the *eGFP* coding region can direct a transcript to the apical cytoplasm. (I and J) Fluorescent in situ hybridization (green) reveals the localization of endogenous mRNAs at stage 10. A probe specific to *sdt* exons 4–6 stains the unlocalized transcripts at stage 10 (J), while a probe specific to exon 3 does not (I). (C–J) The nuclear envelope is marked with WGA (blue). The apical surface is denoted with a dashed line and the basal surface with a solid line. (A) has been adapted with permission from a figure by S. Berger and E. Knust (unpublished).

To better understand the Dynein-dependence and developmental regulation of *sdt* mRNA localization, we mapped the cis-acting signal that targets *sdt* transcripts to the apical cytoplasm using a series of UAS-transgenes (Figure 5B). The *sdt* locus produces several mRNA species that arise from a combination of at least three transcriptional start sites and alternative splicing [9,10,38]. We based our initial constructs on the *sdt A* isoform (also known as *Sdt cMAGUK*), which contains all 10 coding exons in the final transcript. As expected, transcripts from the *sdt A* transgene showed dramatic apical localization in the FCs (Figure 5C). Because the majority of mRNA localization signals reside within the 3′UTR, we replaced the *sdt* 3′UTR on the *sdt A* transgene with the SV40 3′UTR. Surprisingly, this manipulation had no effect on the apical transcript localization (Figure 5D). Similarly, when we appended the *sdt* 3′UTR to an eGFP transgene, *eGFP* transcripts were uniformly distributed in the FC cytoplasm (Figure 5F). These results demonstrate that the *sdt* 3′UTR is neither necessary nor sufficient to act as an apical mRNA localization signal.

In the uncommon cases in which mRNA localization signals lie outside the 3′UTR, they are often found within the coding region [39–43]. Since the majority of *sdt* transcripts are apical at early oogenic stages, but unlocalized at later stages, there must be at least one naturally occurring *sdt* mRNA isoform that lacks the apical signal. The *sdt B* mRNA isoform is identical to *sdt A* except that the third exon is spliced from the final transcript, thus removing 433 amino acids that lie between the two ECR domains (Figure 5A, [10]). Intriguingly, no obvious functional motifs are encoded by exon 3 and this region is absent from vertebrate Sdt homologs (Figure 5A). Given the uncertainty about exon 3′s contribution to Sdt protein function we hypothesized that this exon might contain the apical localization signal for the *sdt* mRNA.

To test this hypothesis we constructed two additional UAS-transgenes and assayed their mRNA localization patterns. The first transgene mimicked the *sdt B* isoform, in that it contained all *sdt* coding exons except exon 3. Transcripts from the *sdt B* transgene are uniformly distributed in the FC cytoplasm (Figure 5E), indicating that exon 3 is required for the apical localization of *sdt* mRNA. Conversely, when exon 3 was appended to the eGFP coding sequence in a second transgene, eGFP transcripts showed dramatic apical localization in the FCs, demonstrating that exon 3 is capable of targeting a normally unlocalized transcript to the apical cytoplasm (Figure 5G–5H). Analogous experiments in embryonic salivary glands gave identical results (Figure S5). These data indicate that exon 3 is both necessary and sufficient for the apical localization of the *sdt* mRNA.

By mapping the *sdt* apical signal to exon 3, our data suggest a model in which the subcellular localization of *sdt* transcripts could be developmentally regulated through pre-mRNA splicing. If this model is correct, the unlocalized, endogenous *sdt* transcripts observed in the FCs at oogenic stage 10 should lack exon 3 and thus correspond to variants of *sdt B*. We designed two exon-specific in situ probes, one that exclusively recognizes exon 3 and a probe of the same size that covers portions of exons 4–6. The ex4–6 probe is predicted to recognize all *sdt* mRNA isoforms, whereas the ex3 probe will reveal all *sdt* isoforms except *sdt B* variants. Although both probes stain localized transcripts in the oocyte with equal intensity (Figure S5), only the ex4–6 probe stains the unlocalized transcripts in the FCs at stage 10 (Figure 5I and 5J). These data suggest that *sdt B* isoforms predominate in the FCs later in oogenesis. Moreover, our results reveal an unusual biological mechanism in which mRNA localization is developmentally regulated through the alternative splicing of a coding exon.

### *sdt A* Is Specifically Required for the Early Formation of Embryonic Epithelia

**Figure 6:**
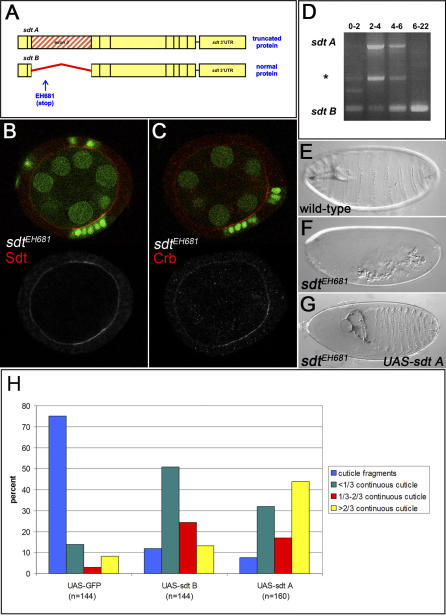
*sdt A* Is Specifically Required for the Early Formation of Embryonic Epithelia (A) The *sdt^EH681^* allele contains a stop codon within the alternatively spliced exon 3. (B and C) Wild-type cells are marked with GFP (green). In *sdt^EH681^* mutant FCs clones, Sdt (B) and Crb (C) are both reduced at the apical surface. (D) Reverse transcriptase-PCR with primers flanking exon 3, showing the relative distribution of *sdt A* and *sdt B* transcripts at different developmental time points in embryos. Asterisk marks a non-specific band. The fragmented cuticle of *sdt^EH681^* embryos (F) when compared to wild-type embryos (E) demonstrates a strong loss of apico–basal polarity in embryonic epithelia. (G) A *sdt^EH681^* mutant embryo in which the *UAS-sdt A* transgene has rescued more than two-thirds of the embryonic cuticle. (H) Graph demonstrating that a *UAS-sdt A* transgene is more efficient at rescuing the *sdt^EH681^* cuticle phenotype than a *UAS-sdt B* transgene or the negative control, UAS-GFP transgene.

To investigate the functional significance of *sdt* transcript localization, we examined the phenotype of *sdt^EH681^*, an allele caused by a premature stop codon within the alternatively spliced exon 3 [10]. In this mutant background proteins translated from apical *sdt A* transcripts should be severely truncated and non-functional [44], while proteins from unlocalized *sdt B* transcripts should be unaffected (Figure 6A). Immunohistochemistry on *sdt^EH681^* FC clones revealed that Sdt and Crb protein levels are reduced at the apical surface of mutant cells (Figure 6B and 6C). These data indicate that Sdt A is required to attain wild-type levels of Sdt and Crb at the apical FCs surface. Interestingly, *sdt^EH681^* cells were morphologically indistinguishable from wild-type, indicating that *sdt* mRNA isoforms lacking exon 3 must also be present in the FCs during early oogenic stages. This supposition is consistent with our previous observation that 26% of *sdt* mRNA puncta are found in the basal cytoplasm during early oogenic stages (Table S1). Together these findings suggest that *sdt A* contributes to the apical targeting of the Crb complex, but that this function is dispensable for FC polarity under normal conditions.

In contrast to the relatively mild phenotype of *sdt^EH681^* in the FCs, this allele produces a very strong phenotype in embryos (Figure 6D and 6E, [36,38]). Since RT-PCR experiments demonstrated that *sdt A* and *sdt B* are both present during early embryogenesis (Figure 6F, [38]), this suggested that apically localized *sdt* transcripts play a significant polarizing role in embryonic epithelia. To test this hypothesis, we compared the ability of *sdt A* and *sdt B* transgenes to rescue the *sdt^EH681^* embryonic phenotype. For this experiment a maternal Gal4 driver ensured early availability of the exogenous mRNAs and the two transgenes were targeted to the same genomic *attP*2 site to provide uniformity in transcript levels. Although both transgenes provide some function, *sdt A* is much more efficient than *sdt B* in rescuing the *sdt^EH681^* phenotype (Figure 6G). Among the embryos collected from the cross in which the *sdt^EH681^* and the *sdt A* transgene were segregating, 43% showed continuous cuticle over greater than 2/3 of their surface. In contrast, only 13% of embryos expressing *sdt B* fell into this class, as compared to 8% in the negative control group. These data suggest that *sdt A* plays a specific and early role in epithelial formation in the embryo.

## Discussion

### Dynein, Apical Determinants, and Polarity

Dynein's role in MT-based apical transport has been studied in the epithelia of both mammals and flies [5,28,45–48], but an explicit link between Dynein and apico–basal polarity has not been found. One cause for this deficiency may be that Dynein is required for a number of essential cellular processes, making it difficult to study this motor under strong loss-of-function conditions. For instance, previous studies of Dynein function in *Drosophila* embryos have relied on combinations of hypomorphic *Dhc* alleles or injection of α-Dhc antibodies which only partially block Dynein function [5,28,33]. Notably, these manipulations have failed to produce epithelial polarity phenotypes. We have shown that strong loss of Dynein function in the FCs disrupts both molecular and morphological aspects of apico–basal polarity, providing the first direct evidence for the role of this motor in this key cell biological process. It is currently unclear why the FCs tolerate strong loss of Dynein function better than other tissues, but this property provides a unique opportunity to begin dissecting the many roles that Dynein is likely to play in epithelial organization.

How does Dynein regulate apico–basal polarity? Since Dynein is a minus-end directed MT motor and minus ends are apically oriented in epithelia, we investigated whether Dynein might ferry components of the Baz and/or the Crb complexes to their sites of action at the apical surface. Two pieces of data indicate that the Baz complex is not the primary Dynein cargo contributing to FC polarity. Although Baz is occasionally relocalized to the lateral FC surface in *Dhc* clones, most mutant cells display significant amounts of apical Baz. Furthermore, ECs in which the entire epithelium is mutant for *baz* or *aPKC* display multilayering at both EC poles ([49], unpublished data), a phenotype that is distinct from the posterior multilayering in *Dhc* mutant epithelia. These observations are consistent with recent studies in the embryonic blastoderm, where it was shown that Dynein does play a role in Baz localization, but that it is not the only means by which this protein is targeted to the apical domain [5]. Together, these data indicate that, while Dynein likely does play a role in the apical targeting of Baz in both the embryo and the FCs, Dynein's major contribution to FC apico–basal polarity corresponds to a different cargo.

Our results favor a model in which Dynein works primarily through the Crb complex to influence epithelial polarity. When Dynein function is reduced in the FCs, Crb and Sdt disappear from the apical surface, even in *Dhc* cells that remain cuboidal. Moreover, the morphology of an egg chamber in which the entire epithelium is mutant for *Dhc* is quite similar to that seen for *crb* and *sdt* mutant egg chambers*,* as all three display multilayering predominantly in the posterior ([31], data not shown). A major challenge comes in deciphering which Crb complex components are specifically transported by Dynein. We have focused on the relationship between Dynein and Sdt because genetic interaction and molecular epistasis experiments indicate that loss of apical Sdt can account for many aspects of the *Dhc* polarity phenotype. Our finding that the apical localization of *sdt* transcripts is Dynein-dependent suggests a mechanism by which Dynein could localize Sdt, in part, through the apical targeting and localized translation of its mRNA. *sdt* transcripts cannot be the only Crb complex component transported by Dynein, however, as the phenotype of *sdt^EH681^* mutant FCs does not recapitulate all aspects of the polarity phenotype of *Dhc* mutant clones. Interestingly, *crb* transcripts are also targeted to the apical FC cytoplasm in a Dynein-dependent manner, although at a later stage than *sdt* (Figure S3). This finding raises the possibility that, in addition to Baz, *crb* mRNA and/or Crb complex proteins represent other Dynein cargoes required for full epithelial polarization. Future work will be required to more finely dissect Dynein's complex contributions to the apical targeting of the Crb complex.

### Developmental Regulation of *sdt* mRNA Localization

While investigating whether *sdt* mRNA was likely to be a primary Dynein cargo contributing to apico–basal polarity, we made the surprising discovery that the apical targeting of *sdt* transcripts is regulated through the alternative splicing of a coding exon, exon 3. We are aware of only two other genes in which transcript localization is regulated in this way [50,51]. In both instances, however, the signal lies within the 3′UTR, so the splicing event does not affect protein structure. It is curious that alternative splicing of the *sdt* mRNA localization signal also deletes 433 amino acids from the protein. The role of the amino acids encoded by exon 3 in Sdt function is not yet known. These amino acids are not conserved in vertebrate Sdt homologs. Moreover, Sdt A and Sdt B bind the intracellular domain of Crb with equal efficiency in vitro [10] and our work with the *sdt^EH681^* allele, as well as over-expression studies with the *sdt A* and *sdt B* transgenes (Figure S1) indicate that both protein isoforms stabilize Crb in vivo. Although we have not ruled out the possibility that exon 3 regulates both mRNA localization and protein function, together these observations suggest that the splicing of exon 3 may primarily regulate mRNA localization.

A potential role for Dynein-dependent apical targeting of *sdt* mRNA in epithelial polarity is supported by our analysis of the relative contributions of the *sdt A* and *sdt B* isoforms. Although the lack of Sdt A in *sdt^EH681^* FC clones reduces Crb at the apical membrane, this deficit leads to relatively mild effects on other aspects of polarity. This apparent discrepancy can be explained by the extrinsic cue for apical identity that is provided to the FCs through their direct contact with the germline [30,31], which may compensate for reduced Crb complex function in this tissue. However, in the embryo, where no such cue is available, *sdt^EH681^* has a nearly null phenotype, which is rescued much more efficiently by *sdt A* than *sdt B.* Overall, these data suggest that the apical targeting of *sdt* transcripts may represent an important mechanism contributing to apico–basal polarity.

Interestingly, the apical targeting of *sdt* mRNA is developmentally regulated in both embryonic and adult epithelia. Specifically, we have shown that apically localized *sdt* transcripts are found only during early stages, while unlocalized transcripts predominate at later stages. Why is *sdt* transcript localization regulated in this way? It is tempting to speculate that apical transcripts are required primarily for the establishment of apico–basal polarity but not its maintenance. In reality, however, the functional distinction between the two phases of *sdt* mRNA localization is almost certainly more subtle. When apical *sdt A* transcripts are present, the epithelia are relatively immature; they tend to be proliferative, display incomplete junctional structures and have yet to adopt their final cell morphology. By contrast, when unlocalized *sdt B* transcripts predominate, the epithelia are more likely to be post-mitotic and highly differentiated. These observations raise the possibility that a concentrated pool of Sdt protein, generated by localized translation of apical transcripts, could function to stabilize Crb primarily during periods when cell polarity is labile, but that this dynamic regulatory mechanism would be dispensable in fully differentiated cells.

## Materials and Methods

### *Drosophila* genetics.

FC clones were generated as previously described [52] using the *e22c-Gal4* driver and *ubiquitin-GFP* to mark wild-type cells. The new *Dhc64C* and *Glued* alleles were created by mutagenizing the FRT80B chromosome with EMS. Other mutant lines used in this study include: *Dhc^4^* ^−*19*^ FRT80B [33], FRT82B *crb^11A22^* [31], *sdt^XP96^* FRT101 [10], *sdt^EH681^* FRT19A [38], and *sdt^P10^* [9]. Gene over-expression studies in FCs were performed using the *GR1-Gal4* driver (generous gift of Trudi Schüpbach) or the hs-FLPout Act-Gal4 driver and the following UAS constructs: *sdt cMAGUK* [9], *nod::lacz* [34] and the 6 transgenes discussed below. Gene over-expression studies in the embryonic salivary gland used the Gal4*^DaG32^* driver [8]. *The Df(3L)Exel6102* and *Df(3L)ED4502* lines are from the Bloomington stock center. All wild-type ovaries and embryos were obtained from OreR flies.

### Immunohistochemistry.

Most ovaries were dissected in PBS and fixed for 15 min at room temperature in 4% EM grade formaldehyde (Polysciences) in PBS. Stainings and washes were performed in PBS with 0.3% triton using the following antibodies: rabbit-α-Baz (1:500, [53] ), rabbit-α-Sdt (1:100, [9]), mouse-α-Dlg 4F3 (1:20, DSHB), rabbit-α-PKCζ C-20 (1:1000, Santa Cruz Biotechnology), and rat-α-Par6 (1:50, [54]). Staining with mouse-α-Crb CQ4 (1:10, generous gift of Eli Knust) was performed with the protocol above, with the addition of a 5 minute methanol wash immediately following fixation. Mouse-α-Dhc P1H4 (1:100) was used as described [55]. Mouse anti-α-Tubulin DM1A (1:500, Sigma) was used as described [35].

### Genetic interaction between *Dhc64C* and *sdt*.

Embryos were collected from the three crosses detailed in Figure 6 over the course of 24 hours. The plates were then aged at 25 °C for an additional 24 hours to allow unaffected embryos to hatch and crawl away. Cuticles were prepared from the remaining mutant embryos. Because *Dhc^4^* ^−*19*^ is larval lethal [33] and the two *sdt* alleles are embryonic lethal, mutant embryos collected were presumed to be *sdt* (−/−). Within the cross segregating both the *sdt^P10^* and *Dhc^4^* ^−*19*^ alleles, the *sdt* (−/−) embryos contain between 0–2 copies of *Dhc^4^* ^−*19*^.

### In situ probe synthesis.

Antisense RNA Probes were synthesized using a DIG RNA labeling kit (Roche). *crb* (LD13435) and *sdt* (RE05272) EST clones were obtained from the *Drosophila* Genome Resource Center (DGRC). The *crb* clone was linearized with NotI and transcribed with the T7 polymerase and the *sdt* clone was linearized with MluI and transcribed with the T3 polymerase. To improve probe penetration, both the *crb* and *sdt* antisense RNAs were fragmented with carbonate buffer. Because the RE05272 EST contains the entire *sdt B* mRNA sequence, *sdt* probe fragments will anneal along the full length of *sdt* transcripts with the exception of exon 3. The antisense eGFP probe was synthesized from the pBS-eGFPA vector, by linearizing with KpnI and transcribing with the T7 polymerase. The ex3 and ex4–6 antisense probes were synthesized by direct transcription from PCR products where the reverse primer included a T7 promoter. Primer sequences can be found in Table S2.

### In situ hybridization.

In situ hybridizations were performed as described in Wilkie (1999) with minor modifications (protocol available upon request). Probes were detected using a horseradish peroxidase conjugated sheep anti-dig antibody (1:1000, Roche) followed by staining with AlexaFluor 488-Tyramide (1:100, Invitrogen). To detect clones in mosaic egg chambers, in situ hybridization was followed by staining with a rabbit anti-GFP antibody and AlexaFluor 555-conjugated secondary antibody (both at 1:200, Invitrogen). The nuclear envelopes and basal lamina were illuminated with AlexaFluor647-conjugated wheat germ agglutinin (1:200, Invitrogen). Images were obtained using a Leica TCS SL confocal microscope and processed with Adobe Illustrator and Photoshop.

### Construction of transgenic animals.

The six transgenes used to map the *sdt* localization signal were cloned into the pUASBP vector (a generous gift from Barret Pfeiffer, Rubin Lab unpublished), which contains the BamHI cassette from pUAST [56] as well as the *attB* element for site-specific recombination using phiC31 integrase [57]. A diagram of the vector and a complete list of primers can be found in Figure S3 and Table S2. *PfuUltra* High-Fidelity DNA Polymerase (Stratagene) was used to amplify *eGFP*, the *sdt 3′UTR*, and *sdt exon3*. All other amplifications used the Phusion High-Fidelity DNA Polymerase (Finnzymes). To create the constructs with the eGFP coding sequence, *eGFP* was amplified from the pBS-eGFPA vector. Directional cloning of this amplicon into pUASBP using 5′ NotI and 3′ XhoI yielded the eGFP-SV40 construct. eGFP-sdt3′UTR was synthesized in two steps. First, the *sdt* 3′UTR was amplified from *sdt cMAGUK* [9]. This amplicon along with pUASBP were digested with XbaI and FseI, gel extracted, and ligated together. Second, *eGFP* was extricated from the eGFP-SV40 construct using NotI and XhoI and subsequently ligated into pUASB containing the *sdt* 3′UTR. For the eGFP-sdtexon3-SV40 construct, exon 3 was amplified from *sdt cMAGUK* and cloned 5′ XhoI and 3′ KpnI into the eGFP-SV40 plasmid. To create the *sdtA*-SV40 transgene, the *sdt* 5′UTR and coding region were amplified from *sdt cMAGUK* and cloned 5′ NotI 3′ XhoI into pUASBP. The *sdt A* construct was then created by cutting the 5′UTR and coding sequence from *sdtA*-SV40 using NotI/XhoI and ligating it into pUASBP containing the *sdt* 3′UTR. The *sdtB* transgene was constructed by amplifying the *sdt* 5′UTR and coding sequence from the RE05252 EST (DGRC) and cloning 5′ NotI and 3′ XhoI into pUASBP containing the *sdt* 3′UTR. Transgenic animals were produced by Genetic Services, who injected DNA for each construct into the *attP*2 line [57] and isolated transformants.

### Reverse transcriptase PCR.

Total RNA was prepped from embryos using TRIzol Reagent (Invitrogen) and first strand cDNA synthesis was performed using the Omniscript reverse transcription kit (Qiagen). Primer sequences can be found in Table S2.

### Rescue of the *sdt^EH681^* mutant phenotype.

Rescue of the *sdt^EH681^* embryonic cuticle phenotype was performed using the maternal P{GAL4-nos.NGT}40 driver [58] and the *UAS-sdt A* and *UAS-sdt B* transgenes. The *UAS-eGFP-sdt 3′UTR* transgene was used as a negative control. Embryos were collected as described for the genetic interaction experiment above.

### Addendum.

A complementary study examining the contributions of Dynein and *crumbs* mRNA localization to apico–basal polarity regulation can be found in the following reference:

Li Z, Wang L, Hays TS, Cai Y (2007) Dynein-mediated apical localization of *crumbs* transcripts is required for epithelial polarity. J Cell Biol. In press.

## Supporting Information

Figure S1 Sdt Stabilizes Crb in the Apical FC Membrane (A) When the entire FC epithelium is mutant for *crb*, Sdt still localizes to the apical surface and junctional region during early oogenic stages, indicating that Sdt is only partially dependent on Crb for its localization in the FCs. This apical localization is gradually lost, however, until Sdt is entirely cytoplasmic by stage 8 (not shown). The bracket denotes Sdt staining in the somatic inner-cyst region.
(B) By contrast, when the entire FC epithelium is mutant for *sdt*, Crb is never seen at the apical membrane, indicating that Crb absolutely requires Sdt for localization.
(C,D) Clonal over-expression of either *sdt A* (C) or *sdt B* (D) increases the amount of Crb in the apical FC membrane at stage 10, a period when endogenous Crb levels in the FC are low. Cells over-expressing the *sdt* transgenes are marked with GFP (green).
(5.8 MB JPG)

Figure S2 Loss of Dhc Has Mild Effects on Baz Complex Localization in the FCs Levels of Baz (A), aPKC (B), and Par6 (C) are mildly reduced at the apical surface of *Dhc^D12^* ^−5^ clones. This apical reduction could be due to the absence of Crb in *Dhc^D12^* ^−5^ clones, as similar reductions are observed in *crb^11A22^* clones (D and F). In occasional *Dhc^D12^* ^−5^ clones Baz and aPKC relocalize to the lateral FC surface (A and B), a phenotype never observed in *crb^11A22^* clones. Wild-type cells are marked with GFP (green) and epithelial gaps are indicated with arrowheads.
(5.8 MB JPG)

Figure S3 Localization of *sdt* mRNA in the Germline and *crb* mRNA in the FCs (A) During early oogenic stages, *sdt* mRNA (green) forms a crescent posterior to the oocyte nucleus, consistent with localization to MT minus ends.
(B) Following the reorganization of the oocyte MTs at stage 7, *sdt* mRNA forms a ring at the anterior of the oocyte. Unlike *sdt* transcripts, which are apically enriched in the FCs during stages 2–4 (C), *crb* mRNA is weakly expressed and uniformly localized during these stages (D). Conversely, at stage 10, when *sdt* transcripts are uniform in the FCs (E), *crb* transcripts are apical (F).
(G,G′) *crb* mRNA is mislocalized in *Dhc* mutant clones (yellow dotted lines) at stage 10.
(A–B,E–G) The nuclear envelope is marked with WGA (blue).
(C–F) The apical surface is denoted with a dashed line and the basal surface with a solid line.
(4.4 KB JPG)

Figure S4 Method for Scoring the Subcellular Location of *sdt* mRNA Puncta (A–C) A single wild-type egg chamber demonstrates the method used to quantify the subcellular location of *sdt* mRNA puncta in the FCs.
(A) Fluorescence in situ hybridization (green) reveals the position of *sdt* mRNA puncta in the egg chamber.
(B) WGA (blue) labels nuclear envelopes and the basal lamina, while an α-Crb antibody (also in blue) marks the apical surface.
(C,C′) The green and blue channels were merged, and each green dot was scored as being within the nucleus (pink), the apical cytoplasm (orange), or the basal cytoplasm (white). For egg chambers in which the entire epithelium was mutant for *Dhc^D12^* ^−*5*^, the position of the apical surface was inferred from GFP expression in the germline (unpublished data).
(D) In situ hybridization on FLPout clones over expressing *UAS-sdt A* (green). Over-expression clones are marked with GFP (red).
(2.5 MB JPG)

Figure S5 Supplemental Data Supporting the Mapping of the *sdt* mRNA Localization Signal (A) Diagram of the pUASBP vector used for transgenic production.
(B,C) Daughterless-Gal4 over-expression followed by in situ hybridization (green) in the embryonic salivary gland reveals that the *sdt* 3′UTR is insufficient to target *GFP* transcripts to the apical surface (B), while *sdt* exon 3 is sufficient (C). White lines indicate the approximate position of the salivary gland basal surface.
(D,E) Control for the specificity of the exon-specific probes. The exon 3 probe (D) and the exon 4–6 probe (E) recognize localized transcripts in the oocyte with equal efficiency.
(B–E) WGA (blue) labels nuclear envelopes.
(3.2 MB JPG)

Table S1 Quantification of the Subcellular Localization of *sdt* mRNA Puncta in Individual Egg Chambers (20 KB DOC)

Table S2 Primer Sequences (35 KB DOC)

### Accession Numbers

Information on *sdt* alleles and sequences for *sdt* mRNA isoforms can be found on Flybase (http://flybase.org/). In this database, *sdt-A* is called *sdt-RB*, and *sdt-B* is called *sdt-RF.*
