# Supplementary material for: Dynein Regulates Epithelial Polarity and the Apical Localization of stardust A mRNA
Source: PLoS Genet. 2008 Jan 18;4(1):e8. doi: 10.1371/journal.pgen.0040008 (PMC2213700; doi:10.1371/journal.pgen.0040008)
Supplement: Supplementary file 1 [file pgen.0040008.st001.doc]

|  |  | **Apical** | **Basal** | **Nuclear** | **% Apical** |
| --- | --- | --- | --- | --- | --- |
| ***wild-type*** | 1 | 58 | 16 | 18 | 78.4 |
|  | 2 | 62 | 19 | 33 | 76.5 |
|  | 3 | 51 | 20 | 15 | 71.8 |
|  | 4 | 62 | 21 | 32 | 74.7 |
|  | 5 | 39 | 12 | 17 | 76.5 |
|  | 6 | 60 | 31 | 26 | 65.9 |
|  | 7 | 31 | 7 | 13 | 81.6 |
|  | 8 | 36 | 14 | 17 | 72.0 |
| ***Dhc^D12-5^*** | 1 | 13 | 16 | 11 | 44.8 |
|  | 2 | 20 | 26 | 15 | 43.5 |
|  | 3 | 12 | 15 | 9 | 44.4 |
|  | 4 | 16 | 27 | 23 | 37.2 |
|  | 5 | 17 | 20 | 22 | 45.9 |
|  | 6 | 24 | 28 | 4 | 46.2 |
|  | 7 | 19 | 19 | 13 | 50.0 |

**Table S1** Quantification of the sub-cellular localization of *sdt* mRNA puncta in individual ECs

The words “apical” and “basal” refer to whether the dot was located in the apical half of the cytoplasm or the basal half of the cytoplasm. The % apical was determined by the following equation: [apical / (apical + basal)] x 100. The wild-type data set has a mean of 74.68 and variance of 22.82, while the *Dhc^D12-5^* data set has a mean of 44.58 with a variance of 14.89. The t value in a student’s t test, therefore, is 13.49, which indicates statistical significance for this result.
