# Supplementary material for: Dynein Regulates Epithelial Polarity and the Apical Localization of stardust A mRNA
Source: PLoS Genet. 2008 Jan 18;4(1):e8. doi: 10.1371/journal.pgen.0040008 (PMC2213700; doi:10.1371/journal.pgen.0040008)
Supplement: Supplementary file 2 [file pgen.0040008.st002.doc]

| **transgenic construction** | |
| --- | --- |
| *sdt* 5’UTR and coding F | TTAGCGGCCGCTAGCGTCAGCGCCGCAGTGT |
| *sdt* 5’UTR and coding R | ATTCTCGAGTTATGACTCGTCGCGATTGTTGTGC |
| *sdt* exon 3 F | TTACTCGAGAAACACAAGAAAAGCACC |
| *sdt* exon 3 R | ATTGGTACCCTCATCGGCCACCCGCC |
| *sdt* 3’UTR F | TTATCTAGATGGGTTCTAAAGCACCCGCAAC |
| *sdt* 3’UTR R | ATTGGCCGGCCTCTCTCTTATGAACGGTTTTATTTC |
| *eGFP* F | TTAGCGGCCGCCGCCACCATGGTGAGCAAGG |
| *eGFP* R | ATTCTCGAGTTACTTGTACAGCTCGTCCATGCC |
|  |  |
| **isoform-specific in situ probes** | |
| *sdt* Ex3 F1 | CGTCCACCACAGAAACACAA |
| *sdt* Ex3 RT7 | TAATACGACTCACTATAGGGAGACCGTCAGCAACACCAGAAAC |
| *sdt* Ex4-6 F1 | AGATCGCCCAGATTGTTGAG |
| *sdt* Ex4-6 RT7 | TAATACGACTCACTATAGGGAGATCTGGATCGTAGTCGAAATGC |
|  |  |
| **RT-PCR** | |
| *sdt* cDNA synthesis | TCACTTTGCTCTTGCATTCG |
| *sdt* Ex3 RT F1 | CATGTTGTCCGTGAACCAAG |
| *sdt* Ex3 RT R1 | AATTGACCAGCCGACCATC |

**Table S2** Primer sequences
